# Potassium Permanganate in Morphine Poisoning

**Published:** 1894-12

**Authors:** 


### Potassium Permanganate in Morphine Poisoning.

Recently opportunity was met with of trying permanganate of potash in a case of morphine poisoning. The patient had swallowed about 60 grains of morphine. The morphine had twelve minutes the start of the antidote. There was only slight drowsiness, but at times threatened narcosis. Ten grains of permanganate was administered in a small glassful of water in three different doses within an hour, using in all about thirty grains of the permanganate, without the least unfavorable effect from its use. In ten hours from the time the morphine was taken, the patient was well. He had no griping, no headache or distress of any kind from the time the antidote was administered until he had entirely recovered from the slight drowsiness. For about an hour after swallowing the poison the patient was kept moving; then he was allowed to lie down and sleep without further trouble.—*Tri-State Medical Journal.*

